# Preparation of High-Transparency Phosphenanthrene-Based Flame Retardants and Studies of Their Flame-Retardant Properties

**DOI:** 10.3390/polym15244665

**Published:** 2023-12-11

**Authors:** Tao Zhang, Yong Liu

**Affiliations:** 1 School of Resource & Environment and Safety Engineering, Hunan University of Science and Technology, Xiangtan 411201, China; a719453323@gmail.com; 2 Work Safety Key Laboratory on Prevention and Control of Gas and Roof Disasters for Southern Coal Mines, Hunan University of Science and Technology, Xiangtan 411201, China; 3 Hunan Provincial Key Laboratory of Safe Mining Techniques of Coal Mines, Hunan University of Science and Technology, Xiangtan 411201, China

**Keywords:** DOPO derivatives, flame-retardant property, epoxy resin, high transparency

## Abstract

Transparency is an important property for polymer flame retardants, especially epoxy resin (EP) flame retardants, and flame-retardant epoxy resins that maintain a high transparency and low chromatic aberration play important roles in the optical, lighting, and energy industries. Herein, a DOPO-based flame retardant 6,6′-((sulfonylbis(4,1-phenylene))bis(oxy))bis(dibenzo[c,e][1,2]oxaphosphinine 6-oxide) with a high transparency and low chromatic aberration was prepared via the classical Atherton–Todd reaction and named SBPDOPO. Its chemical structure was characterized with Fourier IR spectroscopy and NMR spectroscopy. An EP loaded with 7 wt% SBPDOPO passed the UL-94 V-0 rating with an LOI value of 32.1%, and the peak heat release rate, total heat release, and total smoke production were reduced by 34.1%, 31.6%, and 27.7%, respectively, compared with those of pure EP. In addition, the addition of SBPDOPO improved the thermal stability, residual mass, and glass transition temperature of the EP. On this basis, the EP containing 7 wt% SBPDOPO maintained a high transparency and low color aberration, with a transmittance of 94% relative to that of pure EP and a color aberration ΔE of 1.63. Finally, the flame-retardant mechanism of SBPDOPO was analyzed, which demonstrated that it exerted both gas-phase and condensed-phase flame-retardant effects, and that SBPDOPO/EP had high potential for application scenarios in which both flame retardancy and transparency are needed. SBPDOPO/EP has great potential for applications requiring both flame retardancy and transparency.

## 1. Introduction

As some of the most widely used thermosetting resins, epoxy resins (EPs) have good mechanical properties, high transparencies, electrical insulation, excellent chemical stabilities, and easy processing and, therefore, occupy an important position in the coatings, adhesives, electronics, and aerospace fields [1,2,3]. However, EPs are highly flammable and produce molten droplets during combustion, which spread the fire [4,5,6]. Therefore, reducing the flammability of EPs is very necessary.

In recent decades, researchers have used various methods to reduce the fire hazards of EPs. The most common methods include structural modifications, the addition of flame retardants, and the addition of fillers. A structural modification is the introduction of molecules with flame-retardant functions into the structure of the epoxy resin. The modified epoxy resin obtained has long-term effective flame-retardant properties. In addition, the degree of cross-linking and the mechanical properties of the epoxy resin may be improved, influenced by the structure of the introduced molecules. Yuan et al. [7] introduced bifunctional phosphorus-containing triazole derivatives (D-ATAs) into epoxy resins to obtain high-performance epoxy resins with both flame-retardant and anticorrosive properties. Teng et al. [8] prepared a hyperbranched flame retardant (HBFR) containing phosphorus and nitrogen. This flame retardant had a rigid backbone and epoxy groups. The flame retardancy and mechanical properties of the epoxy resin were significantly improved by the incorporation of the HBFR. Ying et al. [9] synthesized an imidazolium diphenylphosphinate hardener (IDPP). The IDPP controls the curing behavior and improves the flame retardancy and thermal stability of epoxy resins. Zhang et al. [10] synthesized a flame-retardant hardener with phosphaphenanthrene, benzothiazole, and imidazole groups (PBIs). It accelerates the cross-linking process of epoxy resins and gives them flame retardancy. The addition of flame retardants is an easy and flexible flame-retardant strategy; flame retardants are blended with the epoxy resin before the curing process and do not participate in the reaction during curing. Currently, popular additive flame retardants mainly include the following: phosphaphenanthrene derivative (DOPO based) [11,12,13], phosphazene derivative [14,15,16], organic phosphate [17,18,19], inorganic phosphate [20,21], nitrogen compound [22,23], silicone compound [24,25], boron compound, biobased flame retardants [26,27,28], composite flame-retardant system [29,30,31,32], etc. The flame-retardant mechanism of the above flame retardants is mainly divided into two aspects: gas phase and condensed phase. The free radical and chain reaction theory is a widely recognized theory in gas-phase flame-retardant mechanisms. During combustion, free radicals continue to take electrons from other molecules and create more free radicals to keep the chain reaction going. The flame-retardant elements in the flame retardant are able to capture these free radicals and interrupt combustion [4,33]. In addition, noncombustible gases such as NH_3_, CO_2_, and water vapor, generated through the combustion of flame retardants, can dilute the combustible and combustion gases in the air [29,34]. For the cohesive-phase flame-retardant mechanism, the main manifestation is the increase in the carbon formation rate. The dense charcoal layer effectively blocks the heat transfer and spillage of combustible volatiles and prevents the direct contact of flames and combustion gases with the epoxy resin’s surface [35,36].

The deterioration of the optical properties of EPs through the addition of flame retardants is relatively common and may be overlooked by researchers. However, in some special fields, such as optical devices, lighting, and photovoltaic power generation industries, transparency may directly affect the performance of the device. Therefore, research on highly transparent flame retardants is of great significance. Currently, researchers have achieved progress in generating highly transparent flame retardants [11,37,38]. Ding et al. [39] prepared the P/S flame retardant SBPDPP from diphenylphosphinyl chloride and 4,4-dihydroxydiphenylsulfone as the raw materials. SBPDPP achieved a UL-94 V-0 grade with a high LOI value of 38.8%, while maintaining a high transparency. Ye et al. [40] prepared a novel hyperbranched polymer (TP) based on trihydroxymethylphosphine oxide and phenylboronic acid as the raw materials, and the TP (2 wt%) increased the LOI of the EP to 33.2%, which reached the UL-94 V-0 grade, and the mechanical properties were also greatly improved. In addition, the TP-doped EP still exhibited a high transparency. Wang et al. [41] prepared a new biobased flame retardant VP from vanillin, 1H-tetrazol-5-amine hydrate, and 9,10-dihydro-9-oxa-10-phosphaphenanthrene 10-oxide (DOPO); at 5 wt%, the VTP achieved a UL-94 V-0 rating for the EP with an LOI value of 30.5%, and maintained a high degree of transparency.

In this work, a P/S-containing flame retardant, 6,6′-((sulfonylbis(4,1-phenylene))bis(oxy))bis(dibenzo[c,e][1,2]oxaphosphinine 6-oxide), was prepared and named SBPDOPO. The chemical structure of SBPDOPO was characterized with Fourier infrared spectroscopy and nuclear magnetic resonance spectroscopy. The flame retardancy of SBPDOPO in an EP was evaluated with thermogravimetric tests, UL-94 tests, LOI tests, and cone calorimetric tests. The flame-retardant mechanism was also proposed by analyzing the residual carbon and volatiles from pyrolysis. Finally, the mechanical and optical properties of the flame-retarded EP were evaluated.

## 2. Experimental Section

### 2.1. Materials

9,10-Dihydro-9-oxa-10-phosphaphenanthrene-10-oxide (DOPO) AR, 4,4′-sulfonyldiphenol(BPS)AR, dichloromethane(DCM)99.5%, carbon tetrachloride (CCl_4_) AR, triethylamine (TEA) AR, and 4,4′-methylenedianiline(DDM)AR were purchased from Shanghai Macklin Biochemical Technology Co., Shanghai, China. Bisphenol A-type epoxy resin (DGEBA, commercial name: E-51; epoxy equivalent weight: 185–208 g/eq; viscosity: 11,000–14,000 mPa·s) was purchased from Nanchang Chenfang Adhesive Products Co., Nanchang, China. Hydrochloric acid (2 mol/L) was self-made in the laboratory.

DOPO is a typical nonhalogenated organophosphorus flame retardant with a biphenyl and phenanthrene ring structure; therefore, DOPO has a high thermal stability, water resistance, and oxidation resistance [5,42]. The P–H bond at the end of DOPO gives it a high reactivity, and it can be reacted with a variety of groups to prepare different derivatives [12]. In this experiment, DDM, as a curing agent with a high transparency, needed to be cured at a high temperature (100–150 °C); the higher thermal stability of DOPO could satisfy this condition, and the antioxidant property of DOPO stops it from being easy to yellow at high temperatures. In addition, the presence of the P–H bond can cause it to react with BPS to obtain a product with a high transparency.

### 2.2. Preparation of Composite Materials

**Scheme 1:**
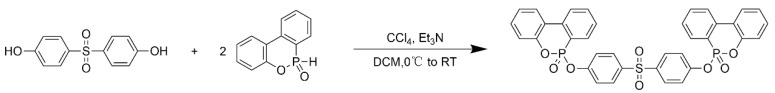
Preparation route of SBPDOPO.

SBPDOPO was prepared with the Atherton–Todd (A–T) reaction. DOPO (8.64 g, 0.04 mol) and BPS (6 g, 0.024 mol) were dissolved in 30 mL of DCM, the mixed system was stirred in an ice-water bath for 10 min, and TEA (4.85 g, 0.048 mol) was slowly added dropwise to the mixed system, which gradually clarified. After the temperature stopped rising, CCl_4_ (7.38 g, 0.048 mol) was added slowly, and the temperature was maintained below 7 °C. Precipitation occurred during the addition. After the dropwise addition, the reaction system was stirred at room temperature for 12 h. At the end of the reaction, the product was washed once with dilute hydrochloric acid and three times with deionized water, and the solvent was removed using distillation under reduced pressure to obtain colorless transparent crystals of SBPDOPO, which were milled to a white powder. The schematic diagram of the synthetic process of SBPDOPO is illustrated in Scheme 1.

E-51 (40 g) and different masses of SBPDOPO were mixed and stirred at 100 °C for 30 min, the temperature of the system was lowered to 89 °C, and DDM (8.5 g) was added, mixed, and stirred for 10 min. The mixture was injected into PTFE molds and placed in a drying oven for curing at 100 °C for 2 h and 150 °C for 3 h. The EP sample formulations with different flame-retardant contents are listed in Table 1.

### 2.3. Characterization

Fourier transform infrared spectroscopy (FTIR) was performed with a Nicolet iN10 Fourier Transform Microinfrared Spectrometer (Thermo Scientific, Waltham, MA, USA) with a wavenumber range of 4000–400 cm^−1^.

P and H nuclear magnetic resonance (NMR) spectra were obtained with an Avance NEO 400 MHz NMR spectrometer (Bruker, Karlsruhe, Germany) in deuterated chloroform.

Thermogravimetric analyses (TGA) were carried out with an STA 449 F3 thermal synchronization analyzer (Netzsch, Selb, Germany). The test atmosphere was nitrogen and the temperature range was 30–800 °C with a heating rate of 10 °C/min.

A vertical combustion test (UL-94) was performed with a VOUCH 5402 horizontal vertical combustion tester (Vouch, Suzhou, China) with ANSI/UL 94.

The limiting oxygen index (LOI) test was performed with an FTT0077 limiting oxygen index meter (FTT, East Grinstead, UK), and the test standard was ASTM D2863 [43].

Cone calorimetry (CONE) was performed with an FTT0007 cone calorimeter (FTT, UK) with sample sizes measuring 100 mm × 100 mm × 3 mm tested according to ISO 5660 [44] and with a radiation intensity of 35 KWm^−2^.

Scanning electron microscope (SEM) photographs were taken with a JSM-6610LV scanning electron microscope (JEOL, Tokyo, Japan). The magnification was 100×.

X-ray photoelectron spectroscopy (XPS) was performed with a K-Alpha X-ray photoelectron spectrometer (Thermo Scientific, Waltham, MA, USA).

Thermogravimetric infrared coupling (TG-IR) was performed with a TG209F1 (Netzsch, Selb, Germany) thermogravimetric analyzer. The heating rate was 10 °C/min and the wavenumber range was 4000–400 cm^−1^.

Dynamic thermomechanical analyses (DMAs) were performed with a TA Q800 (TA, New Castle, DE, USA) dynamic mechanical thermal analyzer. The single cantilever mode was used, the temperature range was 30–800 °C, and the ramp rate was 3 °C/min.

Ultraviolet–visible spectroscopy (UV–VIS) was performed with a UV-3600 spectrophotometer (Shimadzu, Kyoto, Japan). The sample size was 10 mm × 10 mm × 3 mm.

Colorimetry tests were performed with a CS-5960GX spectrophotometer (Jinfulun, Tianjin, China). The sample sizes were 10 mm × 10 mm × 3 mm.

## 3. Results and Discussion

### 3.1. Characterization of SBPDOPO

**Figure 1:**
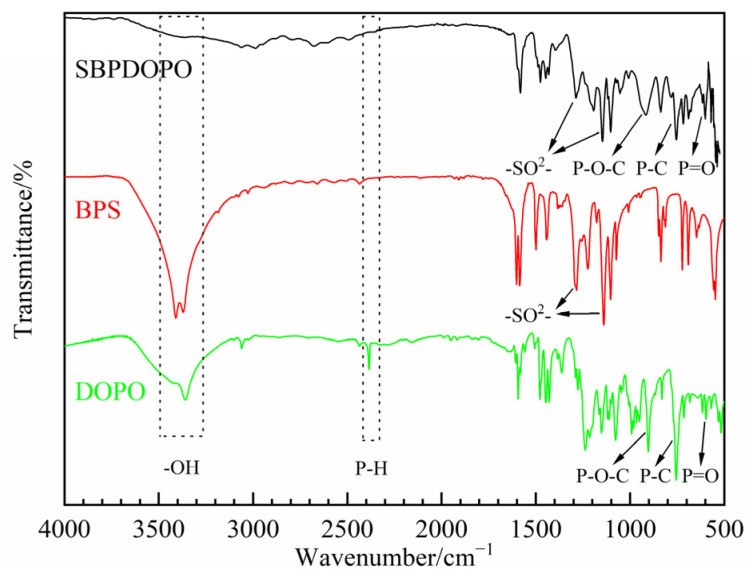
FTIR spectra of DOPO, BPS, and SBPDOPO.

The FTIR spectra of the raw materials DOPO, BPS, and the product SBPDOPO are shown in Figure 1. The peak near 3360 cm^−1^ was attributed to the stretching vibrations of the -OHs; for BPS, the source of -OH was the phenols; additionally, the disappearance of the peak for SBPDOPO at this location indicated that the phenolic hydroxyl group was consumed in the reaction. The peak at 2384 cm^−1^ was attributed to the stretching vibrations of the P-H bonds in DOPO. SBPDOPO did not have a significant absorption peak there, which was due to the reactions of the P-H bonds with the phenolic hydroxyl groups to form P-O-C bonds. The peaks at 1286 cm^−1^ and 1140 cm^−1^ were attributed to the symmetric and antisymmetric stretching vibrations of -SO_2_-, respectively, which were contained in both BPS and SBPDOPO. The peaks appearing near 910 cm^−1^, 755 cm^−1^, and 600 cm^−1^ were attributed to the telescopic vibrational peaks of P-O-C groups, telescopic vibrational peaks of P-C and a telescopic vibrational peak of P=O, respectively, and it can be seen that both DOPO and SBPDOPO exhibited these three peaks, while BPS did not. In summary, in the FTIR spectra of the products, the P-H and phenolic hydroxyl groups disappeared, while the sulfone groups and phosphorus-containing groups were retained, which provided preliminary evidence indicating that the synthesis of SBPDOPO was successful.

**Figure 2:**
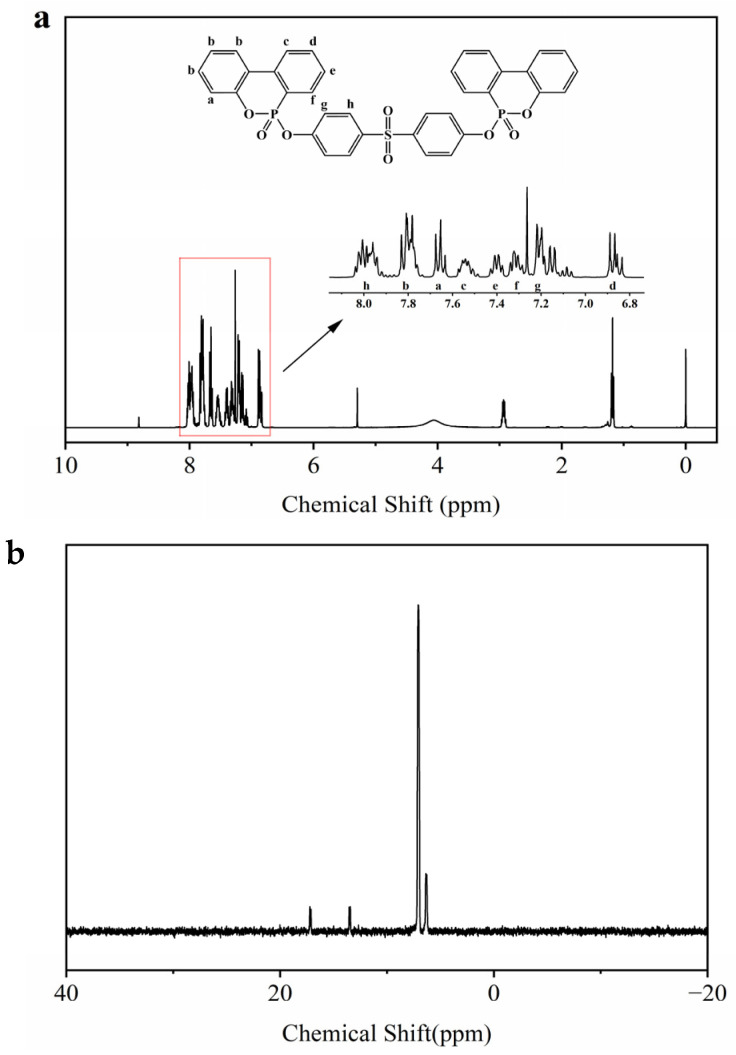
The ^1^H NMR (**a**) and ^31^P NMR (**b**) spectra of SBPDOPO.

The chemical structure of SBPDOPO was verified with ^1^H NMR and ^31^P NMR, and the corresponding spectra are shown in Figure 2. All of the hydrogens were derived from benzene rings, and signals a (7.64, 7.66 ppm), b (7.81, 7.80 ppm), c (7.49, 7.47 ppm), d (6.88, 6.90, 6.85 ppm), e (7.34, 7.36 ppm), and f (7.25, 7.27 ppm) were attributed to the DOPO benzene rings. Signals g (7.18,7.20, 7.14 ppm) and h (7.93 ppm) were attributed to the hydrogens on the benzene ring of BPS. In the ^31^P spectrum, the signals at 6.35 and 7.07 ppm were attributed to phosphorus atoms in SBPDOPO. Combined with the FT-IR results, the successful synthesis of SBPDOPO was confirmed.

### 3.2. Thermal Stability

**Figure 3:**
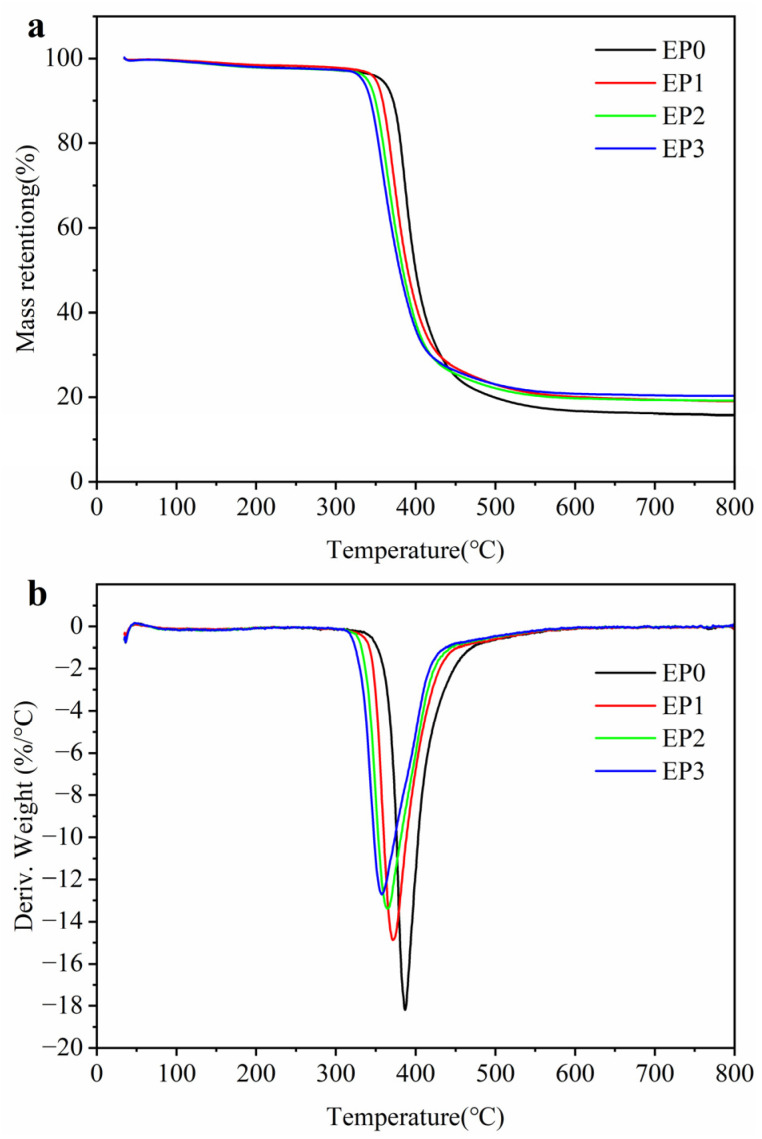
TGA (**a**) and DTG (**b**) curves of EPs with different SBPDOPO contents under nitrogen atmosphere.

To investigate the effects of SBPDOPO on the thermal stability and thermal decomposition process of the EP, TGA studies were carried out under a N_2_ atmosphere. Figure 3 shows the TGA and DTG curves, respectively, and the corresponding data are displayed in Table 1. As shown in Table 2, the corresponding temperature (T5%) for the 5 wt% weight loss decreased as the SBPDOPO content increased, which was due to the catalytic dehydration of the flame retardant. As seen from Figure 3b, in the main pyrolysis stage occurring at 300–500 °C, the addition of SBPDOPO led to a decrease in both the maximum decomposition rate (Rmax) of the EP and the temperatures corresponding to the Rmax and Tmax, which were reduced from 18.18% min^−1^ to 12.72% min^−1^. At this stage, although the flame retardant caused pyrolysis at a lower temperature, it also promoted the cross-linking of the EP into charcoal, and the formation of a charcoal layer slowed the overall decomposition of the material. Moreover, the carbon layer was difficult to decompose at higher temperatures (700–800 °C), so the residue from the EP increased from 15.74% to 20.31%.

### 3.3. Flame-Retardant Properties

To verify the flame-retardant effect of SBPDOPO on the EP, the flame-retardant properties of the EP with different SBPDOPO contents were evaluated with the limiting oxygen index (LOI) and the vertical burning test. The results obtained are shown in Table 3. The table shows that the pure EP had a low LOI value of 24.9%, which would not meet the UL-94 standard, and a serious melt-drop phenomenon, indicating that the untreated EP was highly flammable. With the addition of 3% SBPDOPO, EP1 no longer produced molten droplets, the self-extinguishing time was shortened to a certain extent, and the LOI increased to 29.7%, but it was still unrated. At 5% SBPDOPO, EP2 achieved a UL94 V-1 rating with a further increase in the LOI. EP3 had the highest LOI value of 32.1% and successfully passed the V-0 test with a significant decrease in the average self-extinguishing time, showing good self-extinguishing properties. In addition, EP4 with 3% DOPO also failed the UL94 test, with an LOI of only 27.5, slightly lower than EP1 with 3% SBPDOPO. This may have been attributed to the poor dispersion of DOPO in the EP.

**Figure 4:**
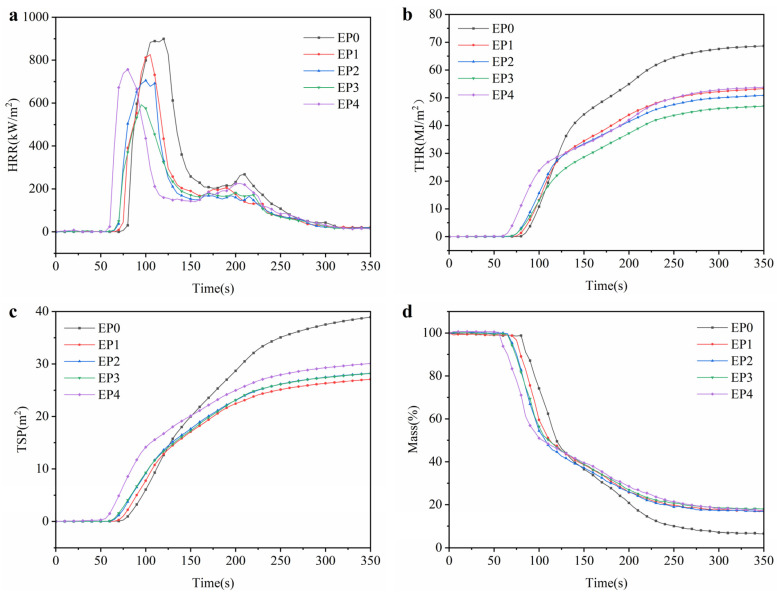
HRR curves (**a**), THR curves (**b**), TSP curves (**c**), and mass loss curves (**d**) for EPs.

The cone calorimetry test (CCT) is widely used with flame-retardant materials. Figure 4 shows the resulting curve for the CCT, including the heat release rate (HRR), total heat release (THR), total smoke production (TSP), and mass loss rate. Table 4 summarizes the test data. The HRR and THR, which are the most important parameters in the CCT, indicate the ability of a material to generate heat during combustion. As shown in the table, the PHRR and THR of EP0 were 899.3 kWm^−1^ and 68.7 MJm^−1^, respectively. The PHRR and THR decreased gradually with the increasing addition of SBPDOPO. Among them, EP3 showed the best effect, with PHRR and THR values of 592.3 kWm^−1^ and 47.0 MJm^−1^, respectively; these represented 34.1% and 31.6% reductions compared with EP0, which indicated that SBPDOPO endowed the EP with excellent flame-retardant properties. In addition, the graphs showed that the time to ignition (TTI) was advanced with increasing SBPDOPO loading, which was due to the decreased crosslink density of the EP and the facilitated decomposition of SBPDOPO, which was consistent with the results of the TG tests. In a fire, the smoke can make escape and rescue difficult and is sometimes more dangerous than the flame itself. Therefore, the TSP of a material is also an important indicator used in assessing the danger of flames. EP0 had a TSP of 39.3 m^2^ at the end of the test, while the smoke generated by the EP treated with the flame retardant decreased dramatically, with TSPs of 27.2, 28.5, and 28.4 m^2^ for EP1, EP2, and EP3, respectively; this indicated reductions of up to 30.8%, which were due to the charcoal-forming effect of SBPDOPO. However, the smoke produced by the EP did not decrease further with the increasing SBPDOPO content, probably because more SBPDOPO hindered the full combustion of the EP, and the decomposition of the initially ignited material into volatiles rich in unsaturated bonds led to a slight increase in the amount of smoke produced and narrowed the TSP gaps among the different samples. In addition, the residual char quality of EP0 was 6.6%, and the residual chars of EP1, EP2, and EP3 were 16.9%, 17.0%, and 17.9%, respectively, indicating that char formation by SBPDOPO was obvious, but the enhancement of the residual char quality was limited by the increased addition. For comparison, cone calorimetry tests were also performed on EP4 containing 3% DOPO. As can be seen from the figure, the PHRR of EP4 was slightly lower than that of EP1 at 756.8 kWm^−1^, while the THR was close to that of EP1 at 53.7 MJm^−1^. The TSP of EP4 was higher than that of all samples doped with SBPDOPO, which was attributed to the fact that DOPO catalyzed the decomposition of the EP prematurely, when the charcoal layer had not yet formed. In addition, the residual mass, CO, and CO_2_ release of EP4 did not differ significantly from the other samples.

### 3.4. Gas-Phase Analysis

**Figure 5:**
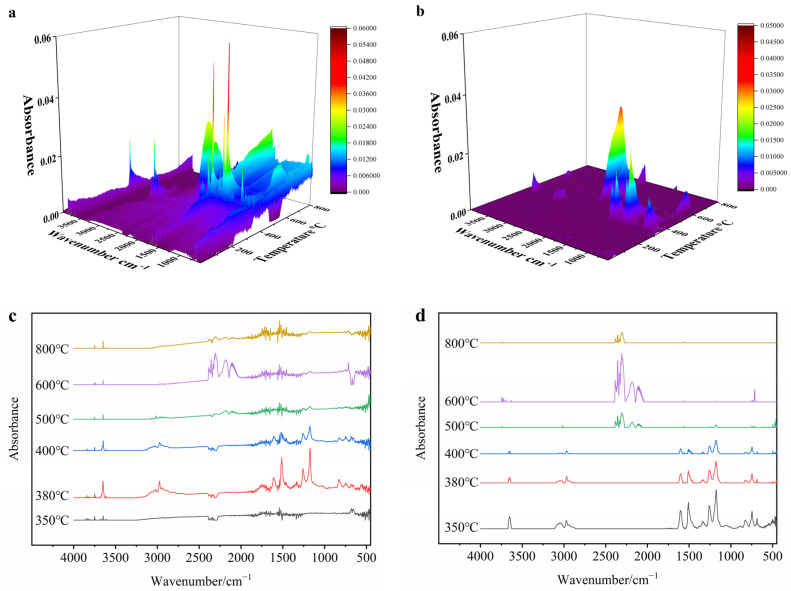
3D images of the pyrolysis products of EP0 (**a**); EP3 (**b**); FTIR spectra of the pyrolysis products of EP0 (**c**); EP3 (**d**) at different temperatures.

To investigate the vapor-phase flame-retardant mechanism of SBPDOPO in the EP, TG-IR studies were conducted with EP0 and EP3. Figure 5 shows the IR spectra of the volatile products formed from SBPDOPO during pyrolysis. The two samples had some identical products, including phenol derivatives (3650 cm^−1^), unsaturated hydrocarbons such as aromatics, olefins, and alkynes (3030 cm^−1^, 3016 cm^−1^, and 2106 cm^−1^, respectively), saturated aliphatic hydrocarbons (2970 cm^−1^, 2936 cm^−1^, and 2884 cm^−1^, respectively), CO_2_, CO (2306 cm^−1^ and 2107 cm^−1^, respectively), benzene rings (1608 cm^−1^, 1512 cm^−1^, and 1330 cm^−1^, respectively), and aromatic and fatty ethers (1259 cm^−1^ and 1170 cm^−1^, respectively). The peaks for the decomposition products were most obvious for heating EP0 at 380 °C, while the decomposition of EP3 was most obvious at 350 °C; this suggested that the addition of SBPDOPO promoted the thermal degradation of the EP, which was consistent with the results of the TG tests. In addition, the two samples also showed differences in their pyrolytic processes, and the peak at 3030 cm^−1^ for the unsaturated hydrocarbons in EP3 was more obvious than that of EP0, which might have been due to insufficient pyrolysis caused by the flame-retardant ability of SBPDOPO. The peaks at 3750 cm^−1^ (H_2_O) and 2306 cm^−1^ (CO_2_) together corroborated this conjecture, and below 400 °C, EP3 hardly produced any H_2_O and CO_2_, suggesting that the oxidation of the EP substrate was inhibited at this temperature. Additionally, EP3 generated a sharp peak at 750 cm^−1^ at this temperature, which was attributed to the phosphorus-containing substances (P-O), indicating that, at this temperature, the phosphorus-containing substances trapped free radicals in the gas phase, which delayed or even terminated the chain reactions of combustion and, ultimately, caused the flame to extinguish itself.

### 3.5. Residual Char Analysis

**Figure 6:**
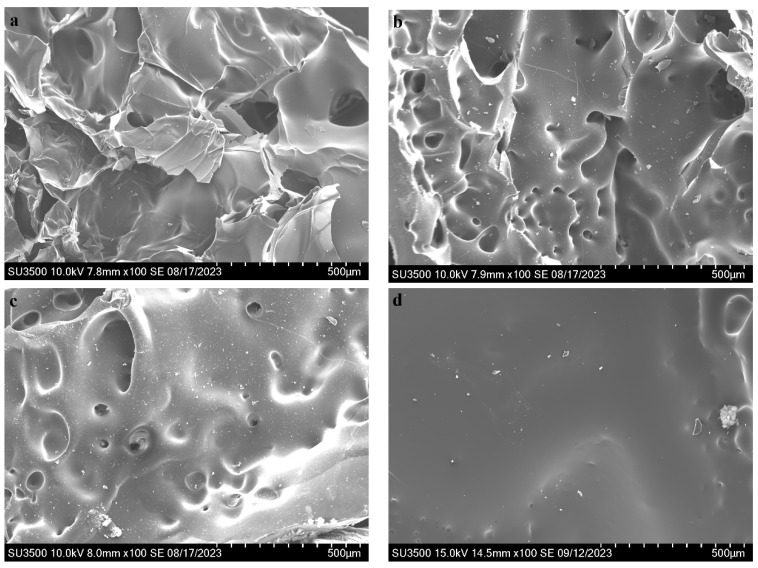
SEM micrographs of the char residues of EP0 (**a**), EP1 (**b**), EP2 (**c**), and EP3 (**d**).

The morphology of the char layer resulting from the UL-94 test was observed via SEM, with the SEM images shown in Figure 6. Apparently, the residual char from EP0 was loose, porous, and accompanied by many fragments; heat, oxygen, and combustibles could easily pass through these voids and cracks, allowing combustion to continue, and the low-strength charcoal layer had difficulty restricting the flow of the molten EP, resulting in the production of molten droplets. With the addition of 3% SBPDOPO, the residual char from EP1 became continuous, but there were still obvious holes and cracks, which were caused by the low strength of the molten char layer, and the gases produced in combustion broke through the char layer. With the increasing SBPDOPO content, the surface of the residual char from EP2 became more complete, the cracks and collapses had almost disappeared, and the number and sizes of the pores decreased significantly. The residual char from EP3 had the most complete morphology, and no visible pores were observed. The intact and dense char layer effectively prevented the heat transfer and limited the spillage of flammable gases from the interior of the material to the surface, resulting in an improved flame retardancy.

**Figure 7:**
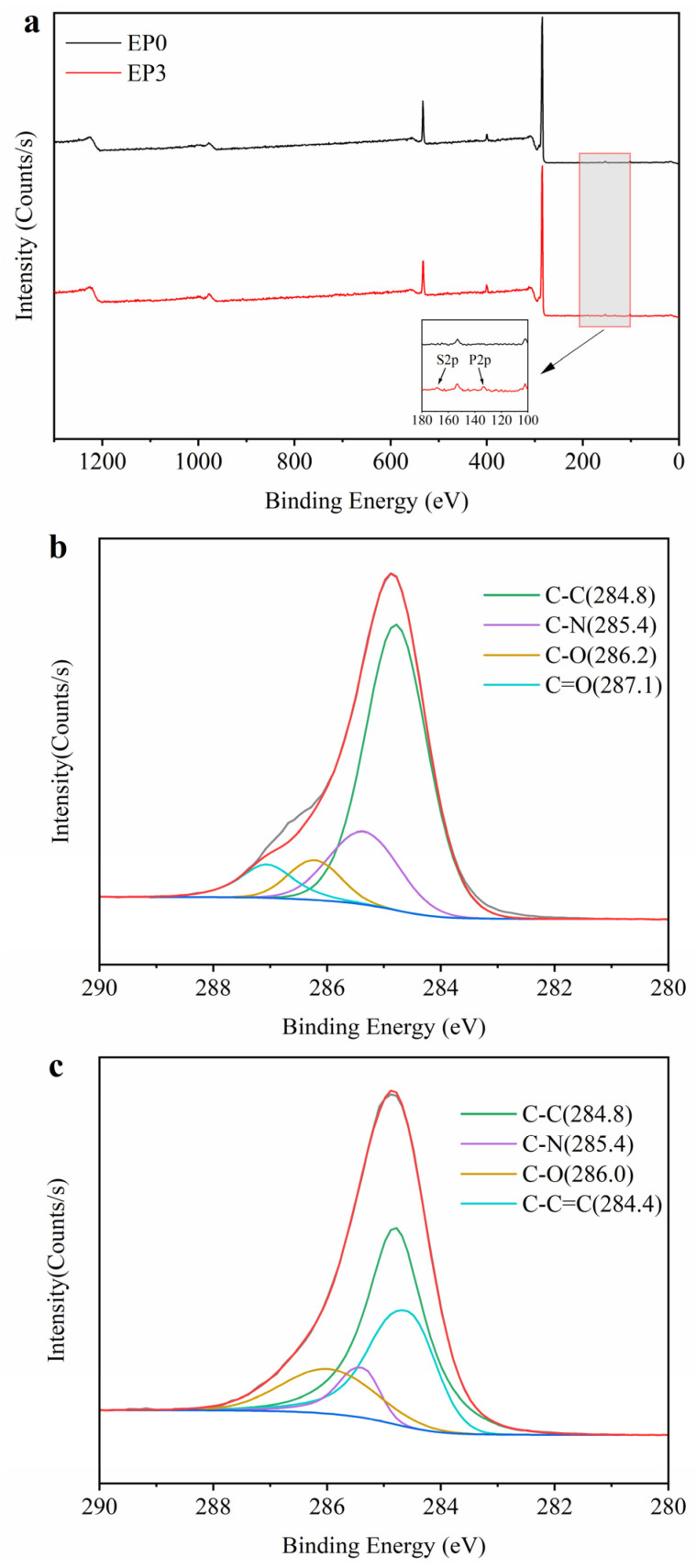

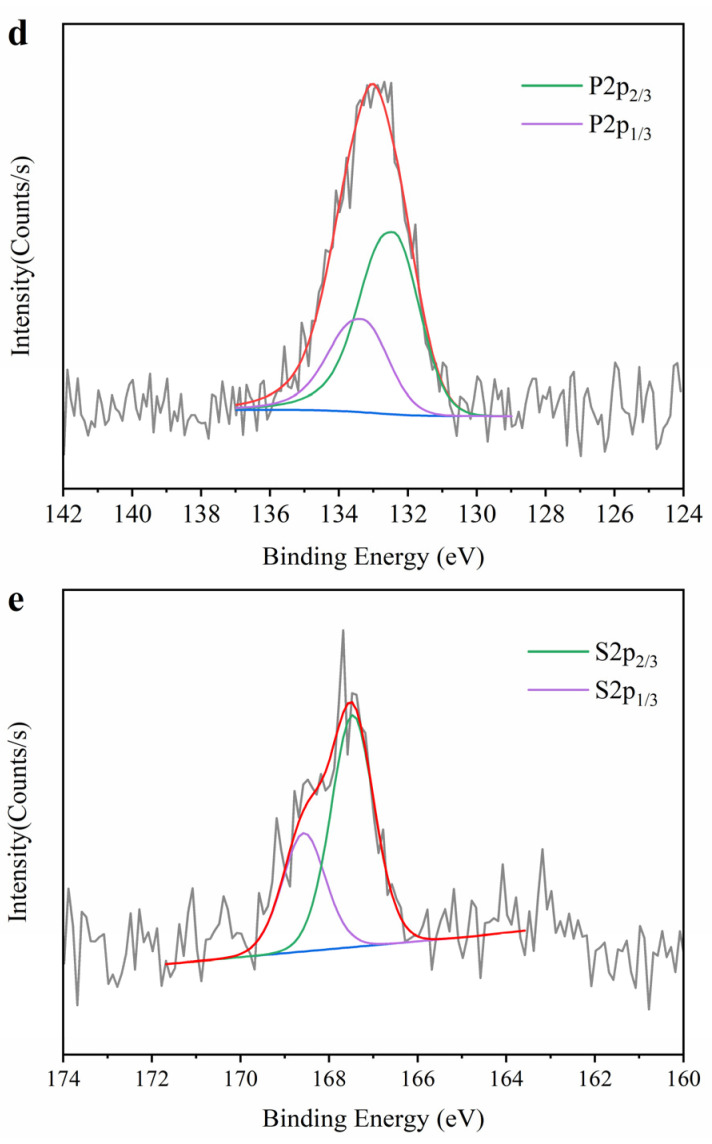
(**a**) XPS spectra of EP0 and EP3; (**b**) EP0-C1s; (**c**) EP3-C1s; (**d**) EP3-P2p; (**e**) EP3-S2p.

XPS studies were performed on the residual chars from EP0 and EP3, with the XPS spectra are shown in Figure 7. Figure 7a shows a comparison of the full spectra for EP0 and EP3. The elements with high contents in both samples were C, O, and N. Due to the low contents of P and S, EP3 showed two weak peaks at 133 eV and 168 eV. The C1s spectra of EP0 and EP3 are shown in Figure 7b,c, respectively. They exhibited the same peaks at 284.4 eV (C-O), 285.4 eV (C-N), and 286.2 eV (C-O), but the disappearance of the peak at 287.1 eV (C=O) for EP3 may have been attributed to reductions in the carbonyl groups due to the catalytic effect of SBPDOPO. In addition, the spectrum for EP3 had a new peak at 284.4 eV (C-C=C), while the peak height at 284.4 eV (C-C) decreased significantly, indicating that the residual carbon of EP3 contained more aromatic compounds; this was attributed to the conversion of the carbonyl chains to aromatic rings due to the catalytic carbon formation through the use of SBPDOPO. Combined with the results from the TG and CCT studies, this showed that SBPDOPO had a more cohesive phase flame-retardant mechanism. Figure 7d shows the P2p spectrum of EP3, and the peak at 133.0 eV was attributed to the P-O-C bonds, which exhibited two spin-orbit peaks with an area ratio of 1:2. The dehydration cross-linking of the phosphate groups in SBPDOPO with EP resulted in a stronger carbon layer, which was less prone to holes and collapse. Figure 7e shows the S2p spectrum of EP3, which contained a peak for the -SO_2_-sulfone groups at 167.5 eV, with two spin-orbit splitting peaks and an area ratio of 1:2. Most of the sulfone groups in SBPDOPO were retained, and the stable nature ensured the strength of the char layer. In conclusion, SBPDOPO promoted the formation of a carbon layer, and most of the P and S were retained in the carbon layer and acted as the skeleton of the carbon layer, which enhanced the density of the carbon layer, blocked the transfer of heat, oxygen, and combustible volatile gases, and exhibited an excellent and cohesive flame-retardant mechanism.

### 3.6. Flame-Retardant Mechanism

**Figure 8:**
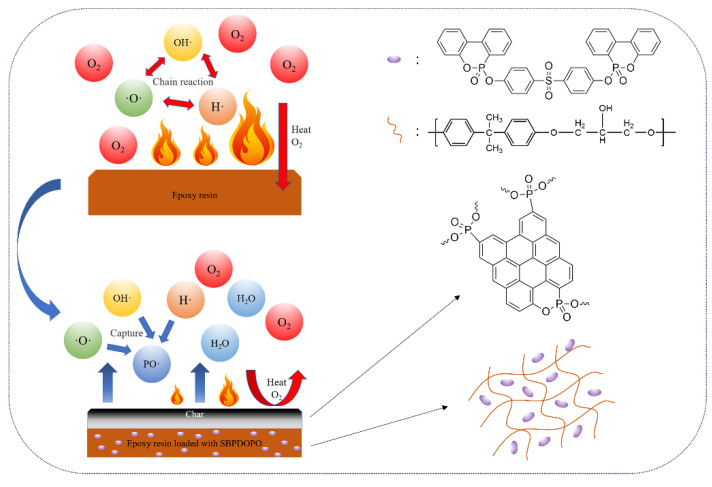
Possible flame-retardant mechanism of SBPDOPO.

As mentioned above, a possible flame-retardant mechanism for SBPDOPO is proposed in Figure 8. SBPDOPO has both gas-phase and condensed-phase flame-retardant mechanisms. In the gas-phase flame retardant, SBPDOPO produced o-phenylphenoxy and phosphorus oxygen groups in combustion; both groups were capable of trapping hydrogen, oxygen, and hydroxyl radicals in the gas phase and producing stable compounds. Thus, the chain reaction was interrupted, further leading to flame bursts. In addition, as can be seen in Figure 5, although SBPDOPO caused combustible gases to be generated earlier, the total amount of combustible gases decreased significantly throughout the combustion process, especially the aromatic compounds and phenol. This meant that SBPDOPO reduced the concentration of flammable gases in the gas phase and utilized the gas-phase flame-retardant mechanism. Furthermore, the water vapor released due to the combustion could also dilute combustible volatiles in the air. In the condensed-phase flame retardant, SBPDOPO was able to catalyze the esterification and dehydration of EP, which resulted in the production of dense and hard residual char. Residual carbon adhering to the EP surface retarded the heat transfer and spillage of combustible volatiles. In the cone calorimetry and thermogravimetry tests, the residual mass of SBPDOPO-doped samples increased, indicating that SBPDOPO could promote the conversion of the EP into a more stable and refractory substance. The dual flame-retardant mechanism of SBPDOPO in the gas phase and condensed phase gave the EP excellent flame-retardant properties.

### 3.7. Mechanical Properties

**Figure 9:**
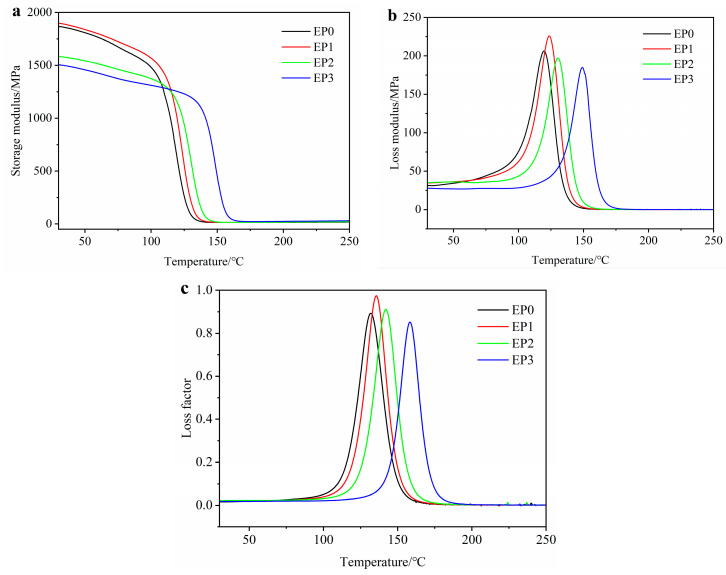
The curves of energy storage modulus (**a**), loss modulus (**b**), and loss factor (**c**).

To investigate the effects of SBPDOPO on the mechanical properties of the EP, DMA tests were conducted on different samples. The curves of the energy storage modulus, loss modulus, and loss factor obtained using the tests are shown in Figure 9. The glass transition temperatures (Tg) of EP0, EP1, EP2, and EP3 were 131.9 °C, 135.5 °C, 142.2 °C, and 158.2 °C, respectively. This indicated that the addition of SBPDOPO improved the Tg of the EP, which was attributed to the higher stiffness and thermal stability of the SBPDOPO itself. In addition, after the addition of 3% SBPDOPO, the storage modulus of the EP at room temperature increased slightly, which may have been due to the increased stiffness caused due to the cross-linking of the residual phenolic hydroxyl groups in SBPDOPO with the EP. At this time, the SBPDOPO content was low, and it did not completely fill the EP network, so it did not cause a significant decrease in the stiffness. With an increasing SBPDOPO content, the energy storage moduli of EP2 and EP3 at room temperature decreased, because SBPDOPO lowered the crosslinking density of the EP during the curing process. Although SBPDOPO reduced the energy storage modulus between the room temperature and the Tg, the energy storage modulus above Tg increased with increases in the content of SBPDOPO, which meant that the EPs with SBPDOPO remained stiff at high temperatures. Therefore, SBPDOPO improved the mechanical properties of the EP at high temperatures and has positive application prospects.

### 3.8. Transparency and Color Aberration

**Figure 10:**
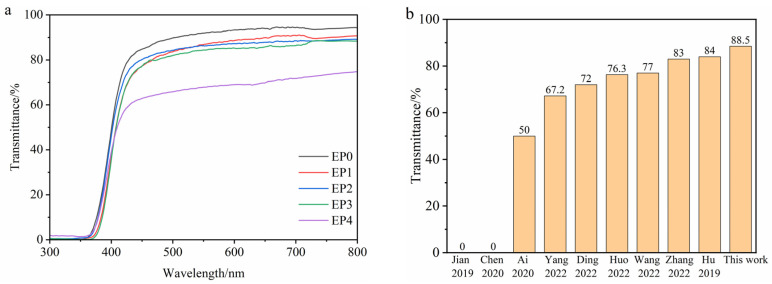
Transparency curves of EPs with different SBPDOPO contents (**a**); comparison for transmission of flame-retardant EP between in this work and other reported references (**b**) Jian2019 [47], Chen 2022 [49],Ai 2020 [50], Yang 2022 [11], Ding 2022 [39], Huo 2022 [48],Wang 2022 [46], Zhang 2022 [45], Hu 2019 [22].

To investigate the effect of the added SBPDOPO on the transparency of the EP, the transparencies of different samples were tested with ultraviolet–visible spectroscopy (UV–VIS), and the curves in Figure 10a show the variations in transmittance with wavelength. The figure shows that all of the samples exhibited a high transparency at visible wavelengths (390–780 nm). The transparencies of the samples decreased slightly with the increasing SBPDOPO contents. At 780 nm, the transmittance of EP0 was 94.1%, and the transmittances of EP1, EP2, EP3, and EP4 were 90.4%, 89.1%, 88.5%, and 74.7%, respectively, which were 3.7%, 5.0%, 5.6%, and 20.6% lower, respectively, than that of EP0. This indicated that SBPDOPO had a minimal effect on the transparency of the epoxy resin. However, DOPO is a white opaque powder and is also less compatible with EPs, thus, significantly reducing the transparency of EP4.

In addition, in order to visualize the advantages of this work in terms of transparency, we compared SBPDOPO/EP with other literature works [11,22,39,45,46,47,48,49,50]. As shown in Figure 10b, in comparison with reference works, the EP doped with SBPDOPO had the highest transparency.

**Figure 11:**
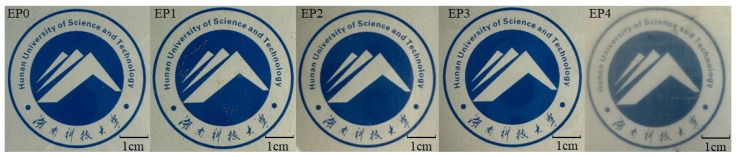
The digital photos of EPs with different SBPDOPO contents.

Figure 11 shows digital photographs of different EP samples covered with patterns; it can be seen that there was a slight deepening of the EP color as the SBPDOPO content rose. However, it was hard to tell with the naked eye, except for EP4 containing 3% DOPO. To further investigate the effect of the added SBPDOPO on the color of the EP, a spectrophotometer was used to carry out chromaticity tests with different samples; the resulting chromaticity data are shown in Table 5, where L*, a*, and b* represent the light/dark, red/green, and yellow/blue differences between the samples and the standards, respectively, and the chromaticity differences ΔL*, Δa*, and Δb* between EP1, EP2, and EP3, respectively, with respect to EP0 were calculated from them. Finally, the total aberration of color ΔE was calculated with Formula 1. It is generally believed that when ΔE < 1, it is difficult for the human eye to recognize color aberrations; when 1 < ΔE < 3.3, they can be recognized by professionally trained people, and when ΔE > 3.3, it is easy to recognize the color aberrations [51]. These results showed that the ΔE values of EP1, EP2, and EP3 relative to EP0 were 1.60, 1.06, and 1.63, respectively. It was assumed that the effect of SBPDOPO on the color of the EP was minimal.

The minimal effect of SBPDOPO on both the transparency and color of the EP may have been due to the following two reasons: First, the transparency of the diphenyl sulfone (DS) groups. 4,4′-diaminodiphenylsulfone (DDS), BPS, and other substances with DS structures are raw materials for the preparation of highly transparent EPs, and BPS and diphenyl phosphoryl chloride (DPPC) have been cited as the raw materials used to prepare highly transparent phosphorus and sulfur-containing flame retardants. Similarly, SBPDOPO with a DS structure exhibits excellent transparency and extremely high compatibility with EPs. Second, SBPDOPO has a high thermal stability. Since the DDM needs to be cured at medium or high temperatures (100–150 °C), some phosphorus-based flame retardants may decompose or undergo side reactions at this temperature, which tends to deepen the color of the EP. It is worth mentioning that the P–H bonds of DOPO also participated in the curing reaction of the EP, so the direct addition of DOPO would also affect the color of the EP. On the other hand, the preparation process for SBPDOPO transformed the P-H bond into a P-O-C bond, and the P element was more stable, so SBPDOPO had little effect on the color of the EP. Therefore, SBPDOPO, which has a high transparency, low color aberration, and high flame retardancy at the same time, has great potential for use in specific application scenarios.

## 4. Conclusions

A highly transparent flame retardant, SBPDOPO, was prepared using the Atherton–Todd reaction. The FT-IR and NMR results indicated that SBPDOPO was synthesized successfully. The TG tests showed that SBPDOPO improved the thermal stability of the EP, with a decrease in the peak pyrolysis rate, a slight increase in the temperature it corresponded to, and an increase in the residual mass. Compared with pure EP, the addition of 7 wt% SBPDOPO enabled the EP to pass the UL-94 V-0 rating with an LOI value of 32.1%, and the peak heat release rate, total heat release, and total smoke production were reduced by 34.1%, 31.6%, and 27.7%, respectively, compared with those of pure EP. Furthermore, the flame retardancy of the sample containing 3% DOPO was slightly lower than that of 3% SBPDOPO. This showed that SBPDOPO could reduce fire hazards resulting from both heat release and smoke. The volatiles formed in the combustion of the EP with the addition of SBPDOPO were analyzed with TG-IR, and it was found that SBPDOPO reduced the products of complete EP combustion and produced phosphorus-containing substances, indicating that elemental phosphorus played a role in the gas-phase flame-retardant mechanism. The morphology and chemical states of the EP residual char were tested with SEM and XPS, and it was found that SBPDOPO enhanced the strength of the char layer through the formation of a crosslinked structure, which hindered the diffusion of heat, oxygen, and volatile combustibles and exhibited a cohesive flame-retardant mechanism. In addition, SBPDOPO increased the Tg of the EP, which increased the strength of the material at high temperatures, but had a negative effect on the energy storage modulus of the material at room temperature. Finally, the effects of SBPDOPO on the transparency and color aberration of EPs were evaluated, and the results showed that 7 wt% SBPDOPO reduced the transparency of the EP by only 5.6%, and the resulting color aberration was difficult to discern with the naked eye. As a comparison, the transparency of EP decreased significantly after 3% DOPO was added. This work gave EPs excellent flame-retardant properties, while ensuring a high transparency and low color aberration, and the amount of flame retardant added was small, which has potential application value.

## Figures and Tables

**Table 1:** Formulas of the cured EP thermosets.

Samples	E-51 (g)	DDM (g)	SBPDOPO (g)	DOPO (g)	P (wt%)
EP0	40	9	0	0	0
EP1	40	9	1.52	0	0.27
EP2	40	9	2.58	0	0.46
EP3	40	9	3.69	0	0.64
EP4	40	9	0	1.52	0.43

**Table 2:** TGA and DTG data for EPs with different SBPDOPO contents.

Samples	T_5%_/°C	T_max_/°C	Rmax/%min^−1^	Residue/%
EP0	357.2	386.9	18.18	15.74
EP1	349.7	371.7	14.88	19.02
EP2	339.2	364.9	13.39	19.16
EP3	333.2	357.7	12.72	20.31

**Table 3:** Combustion test data for EPs with different SBPDOPO contents.

Samples	Average-t_1_/s	Average-t_2_/s	Dripping (Y/N)	Ignite Absorbent Cotton (Y/N)	Rating	LOI/%
EP0	>30	/	Y	Y	NR	24.9
EP1	27.8	7.7	N	N	NR	29.7
EP2	8.7	4	N	N	V-1	30.9
EP3	3.1	4.1	N	N	V-0	32.1
EP4	29.2	31.7	N	N	NR	27.5

**Table 4:** CCT data of EPs with different SBPDOPO contents.

Samples	THR	PHRR	TSP	Residue/%	av-COY	av-CO_2_Y
EP0	68.7	899.3	39.2	6.6	0.12	1.32
EP1	53.3	825.9	27.2	16.9	0.14	1.24
EP2	51.0	705.4	28.4	17.0	0.16	1.23
EP3	47.0	592.3	28.3	17.9	0.17	1.21
EP4	53.7	756.8	30.1	17.3	0.17	1.25

**Table 5:** Colorimeter data of EPs with different SBPDOPO contents.

Samples	L*	ΔL*	a*	Δa*	b*	Δb*	ΔE
EP0	93.72	/	−1.13	/	4.78	/	/
EP1	94.80	1.08	−1.79	−0.49	5.86	1.08	1.60
EP2	94.78	1.06	−1.36	−0.06	4.85	0.07	1.06
EP3	94.15	0.43	−1.74	−0.44	6.29	1.51	1.63

## Data Availability

The data that support the findings of this study are available.
